# Prevalence of the metabolic syndrome and its components in relation to socioeconomic status among Jamaican young adults: a cross-sectional study

**DOI:** 10.1186/1471-2458-10-307

**Published:** 2010-06-03

**Authors:** Trevor S Ferguson, Marshall K Tulloch-Reid, Novie OM Younger, Jennifer M Knight-Madden, Maureen Samms-Vaughan, Deanna Ashley, Jan Van den Broeck, Rainford J Wilks

**Affiliations:** 1 Epidemiology Research Unit, Tropical Medicine Research Institute, The University of the West Indies, Mona, Kingston, Jamaica; 2 Sickle Cell Unit, Tropical Medicine Research Institute, The University of the West Indies, Mona, Kingston, Jamaica; 3 Department of Obstetrics, Gynaecology and Child Health, The University of the West Indies, Mona, Kingston, Jamaica; 4 Institute for Sustainable Development, The University of the West Indies, Mona, Kingston, Jamaica; 5 Centre for International Health, University of Bergen, Bergen, Norway

## Abstract

**Background:**

The metabolic syndrome has a high prevalence in many countries and has been associated with socioeconomic status (SES). This study aimed to estimate the prevalence of the metabolic syndrome and its components among Jamaican young adults and evaluate its association with parental SES.

**Methods:**

A subset of the participants from the 1986 Jamaica Birth Cohort was evaluated at ages 18-20 years between 2005 and 2007. Trained research nurses obtained blood pressure and anthropometric measurements and collected a venous blood sample for measurement of lipids and glucose. Prevalence of the metabolic syndrome and its components were estimated using the 2009 Consensus Criteria from the International Diabetes Federation, National Heart Lung and Blood Institute, American Heart Association, World Heart Federation, International Atherosclerosis Society, and International Association for the Study of Obesity. SES was assessed by questionnaire using occupation of household head, highest education of parent/guardian, and housing tenure of parent/guardian. Analysis yielded means and proportions for metabolic syndrome variables and covariates. Associations with levels of SES variables were obtained using analysis of variance. Multivariable analysis was conducted using logistic regression models.

**Results:**

Data from 839 participants (378 males; 461 females) were analyzed. Prevalence of the metabolic syndrome was 1.2% (95% confidence interval [95%CI] 0.5%-1.9%). Prevalence was higher in females (1.7% vs. 0.5%). Prevalence of the components [male: female] were: central obesity, 16.0% [5.3:24.7]; elevated blood pressure, 6.7% [10.8:3.3]; elevated glucose, 1.2% [2.1:0.4]; low HDL, 46.8% [28.8:61.6]; high triglycerides, 0.6% [0.5:0.6]. There were no significant differences in the prevalence of the metabolic syndrome for any of the SES measures used possibly due to lack of statistical power. Prevalence of central obesity was inversely associated with occupation (highly skilled 12.4%, skilled 13.5%, semi-skilled/unskilled 21.8%, p = 0.013) and education (tertiary 12.5%, secondary 14.1%, primary/all-age 28.4%, p = 0.002). In sex-specific multivariate logistic regression adjusted for hip circumference, central obesity remained associated with occupation and education for women only.

**Conclusion:**

Prevalence of the metabolic syndrome is low, but central obesity and low HDL are present in 16% and 47% of Jamaican youth, respectively. Central obesity is inversely associated with occupation and education in females.

## Background

The metabolic syndrome has emerged as an important clinical entity over the last two decades following Raeven's description of a clustering of risk factors for coronary artery disease in 1988 [1]. The specific components of the metabolic syndrome include: central obesity, glucose intolerance, elevated triglycerides, low levels of high density lipoprotein cholesterol (HDL), and hypertension [2]. The components occur together more frequently than expected by chance, and when grouped together they result in an increased risk for cardiovascular disease and diabetes mellitus [2,3].

Several organizations have published diagnostic criteria for the metabolic syndrome [4-9]. In 2005 the American Heart Association and the National Heart Lung and Blood Institute (AHA/NHLBI) and the International Diabetes Federation (IDF) published criteria [5,6] for the diagnosis of the metabolic syndrome which were designed to have practical application in epidemiological studies. The AHA/NHLBI criteria were essentially a revision of the 2001 National Cholesterol Education Programme (NCEP) Adult Treatment Panel III (ATP-III) criteria [4]. Both the IDF and the revised ATP-III used the five components mentioned above but differed in that the IDF criteria regarded central obesity as a required component with the diagnosis being made if any two of the other components were present, while the revised ATP-III criteria could be based on any three of the five components. The IDF also recommended ethnic specific cut-points for central obesity while the revised ATP-III criteria used just one set of cut-points. More recently both groups met with other representatives from the World Heart Federation, International Atherosclerosis Society, and International Association for the Study of Obesity and published a revised set of criteria [10] in order to harmonize the definition of the metabolic syndrome. These "consensus criteria" again included the same five components, but did not designate any component as required and recommended the use of ethnic or country specific cut-points for central obesity.

Worldwide, prevalence estimates for the metabolic syndrome in men range from eight percent in India to 24 percent in the United States and for women from seven percent in France to 46 percent in India [11]. Prevalence estimates varied with sex, age and ethnicity [12,13]. There are limited published data on the prevalence of the metabolic syndrome among adolescents and young adults. Prevalence estimates among youth range from one percent among young men with mean age of 15 years in Japan, 6.4 percent among adolescents 12-19 years old in the United States, 6.5 percent among adolescents 10-18 years old in Mexico and 10 percent among adolescents 10-19 years old in Iran [14-17] while among US black youth 12-19 years old it is estimated at 4.0% [18]. Although the absolute CVD risk associated with the metabolic syndrome in youth is low it has been shown that clusters of risk factors track into adulthood even more than single risk factors [19,20], suggesting that early identification may be beneficial in the amelioration of the diseases associated with this syndrome.

There are no published data on the prevalence of the metabolic syndrome among youth in Jamaica or other countries of the English-speaking Caribbean. In one study from Trinidad and Tobago 32% of first year medical students 18-23 years old had at least one metabolic syndrome component but the overall metabolic syndrome prevalence was not reported [21].

The role of socioeconomic status in cardiovascular disease epidemiology has been well established [22]. In developed countries most studies report an inverse relationship between socioeconomic status and cardiovascular disease; however the relationship is less clear in low and middle income countries, with some studies showing a positive relationship and others a non-linear association [23,24]. A number of studies have reported associations between the prevalence of the metabolic syndrome and socioeconomic status [25-27]. There appears to be an inverse relationship between socioeconomic status and prevalence of the metabolic syndrome with a stronger association for women compared to men [25,26,28-30].

To date, data on the prevalence of the metabolic syndrome in developing countries is quite limited despite its association with cardiovascular diseases and the current epidemic of the latter in such countries [31]. This paper will report on the prevalence of the metabolic syndrome, and evaluate its association with markers of socioeconomic status in a sample of young adults 18-20 years old, in Jamaica, a middle income, developing country with a predominantly black population.

## Methods

### Participants and recruitment

The authors conducted a cross-sectional study using a sub-set of participants from the 1986 Birth Cohort of the Jamaica Perinatal Mortality Survey [32]. Participants were interviewed and examined between March 2005 and February 2007 and were 18-20 years old at the time of enrolment.

The original participants comprised 10,310 consecutive births in all fourteen parishes in Jamaica during September and October 1986. A sub-sample of participants from three parishes (Kingston, St. Andrew and St. Catherine) was re-evaluated at age 11-12 years [33] and again at age 15-16 years. Follow up studies were limited to these three parishes due to funding limitations. These three parishes account for approximately 43% of the island's population. For the current study the authors obtained contact information for persons seen in the last follow-up (1565 persons) and another 69 persons from the cohort who were identified using information from schools and colleges in the three parishes. Successful contact was made with 1212 participants of whom 902 were enrolled in the study.

Participants were recruited primarily through telephone contact using the last known telephone number or through visits to the last known home address. Eligibility was confirmed by date of birth and address. Those who agreed to participate were given appointments and subsequently transported to the research institution after a 10 hour overnight fast. The study protocol was approved by the University Hospital of the West/University of the West Indies/Faculty of Medical Sciences Ethical Committee. The Ethics Committee of the London School of Hygiene and Tropical Medicine also approved this study. All participants provided written informed consent.

### Measurements

Trained research nurses obtained measurements of blood pressure and anthropometry (weight, height, waist and hip circumference). Blood pressure was measured using a mercury sphygmomanometer following standardized procedures developed for the International Collaborative Study of Hypertension in Blacks [34]. Waist and hip circumferences were measured using a non-stretchable nylon tape. Height was measured using a portable stadiometer and weight was measured using a portable digital scale which was calibrated daily using standard weights. Questionnaires were administered by trained research nurses during face to face interviews. Data on personal and family medical history, socioeconomic status, physical activity and habits were collected. A fasting blood sample was obtained by venepuncture to measure fasting glucose, triglycerides and high density lipoprotein cholesterol. Glucose was measured using the glucose oxidase method (Alcyon Analyzer), while HDL-cholesterol and triglycerides were measured directly using enzymatic techniques (Abbot Spectrum Analyzer).

Physical activity levels were derived based on time spent playing sports or engaged in exercise (such a brisk walking) during leisure time. Participants reporting no leisure time physical activity were classified as having low physical activity level, participants reporting engagement in physical activity of 3.5 hours or less were classified as moderate of physical activity level and those reporting greater than 3.5 hours of physical activity as high physical activity level.

### Measurement of socioeconomic status

Socioeconomic status was assessed using the following measures obtained from the questionnaire: occupation of household head, highest educational level attained by either parent or guardian, and housing tenure of parents/guardian.

Information on education was collected as the highest level of parental education attained using the following categories: never attended school, basic school only, primary/all-age, secondary/high/technical and tertiary-college/university. None of the respondents reported 'no education' or 'basic school education' as the highest parental education level; categories were therefore presented as: primary/all-age; secondary and tertiary. Persons who responded "don't know" to the questions on education of parents/guardian were treated as a fourth category.

Information on occupation was collected as the household head's usual occupation and coded according to the Jamaica Standard Occupational Classification 1991(JSOC-91) [35]. JSOC-91 places occupations in ten major groups in accordance to the International Standard Classification of Occupations 1988 [36]. For this analysis participants were placed into three groups: highly skilled (JSOC-91 groups 1-3), skilled (JSOC-91 groups 4-7), and semi-skilled/unskilled (JSOC-91 groups 8-9). The JSOC-91 places persons in the armed forces in group 10; however, for this analysis they were included in category five along with police officers and other service workers. Persons classified as housewives, students, those retired with no information on last occupation or persons whose stated occupation could not be classified were placed in a fourth group called "Other".

Data on housing tenure of the participants' home was collected in the following categories: owned by parent/guardian, owned by other family member, rented or 'other'. Cases reported as 'other' were generally owned by relatives who were not part of the household or by friends of the family.

### Metabolic syndrome designation

Metabolic syndrome and abnormal values for the individual components were defined using the 2009 Consensus Criteria proposed by the IDF, AHA and others [10]. Participants were classified as having the metabolic syndrome if they had any three of the following five components: elevated waist circumference (men ≥ 94 cm, women ≥ 80 cm); elevated triglycerides (> 1.7 mmol/L); reduced high density lipoprotein (HDL) cholesterol (men < 1.0 mmol/L, women < 1.3 mmol/L); elevated blood pressure (systolic ≥ 130 mmHg, diastolic ≥ 85 mmHg) or elevated fasting glucose (≥ 5.6 mmol/L). Hypertension was defined as having a systolic blood pressure ≥ 140 mmHg or diastolic blood pressure ≥ 90 mmg or being on medication for hypertension. Diabetes was defined as having a fasting glucose ≥ 7.0 mmol/L. None of the participants were currently on medication for hypertension, elevated triglycerides or reduced HDL while only one participant was on medication for diabetes mellitus. The participant who was on medication for diabetes also had a high fasting glucose. The designation of metabolic syndrome or elevated level of the individual component was therefore based on measured values as no additional persons were identified by being on treatment for any of the conditions.

### Statistical methods

Data from questionnaires and laboratory reports were entered into an electronic database using EpiData 3.1. Range and consistency checks were used to minimize data entry errors. Data analysis was done using STATA 9.2 [37]. Mean values and prevalence estimates for the various metabolic syndrome components were obtained within and across sex and socioeconomic status groups. Differences in proportions for categorical variables were compared using χ^2^ tests or Fisher's exact test as appropriate. Tests for trend in the proportions were done across the SES groups excluding the 'don't know' and 'other' groups as it is uncertain where to place these groups in the ordinal range. One-way analysis of variance (ANOVA) was used to compare means of continuous variables across multiple subgroups. If there was unequal variance between subgroups for the ANOVA tests, the Kruskal-Wallis test was used to compare the groups.

Multivariate analyses were carried out using logistic regression. Adjusted odds ratios for the presence of the metabolic syndrome and each of its individual components were obtained separately for each of the three socioeconomic status indicators. Initial models included sex, age, physical activity levels. BMI was also included in models which did not include central obesity. The likelihood ratio test was used to evaluate whether each variable included in the model resulted in a significant improvement of the model. Interaction terms for sex were added to the models, and if found to be significant sex specific odds ratios were presented. Adjustments were also made for hip circumference in models with central obesity in light of studies which suggest that waist and hip circumference have separate effects on CVD risk [38,39].

## Results

The basic characteristics of the participants in the study are shown in Table 1. Analysis was based on data from 839 participants (378 males and 461 females) with complete information on the variables of interest. Sixty-three participants with missing data were excluded. Mean ages were similar in males and females. Males were taller and heavier than females. Females had higher mean body mass index but this did not achieve statistical significance. Similarly, the higher mean waist circumference in males was not statistically significant but systolic and diastolic blood pressures and fasting glucose were higher in males. Females had higher mean high density lipoprotein cholesterol (HDL) and males higher fasting triglycerides. There were no differences in the mean values for the variables described above for participants excluded from the analysis compared to those included.

**Table 1:** Basic characteristics of participants in study by sex

Variables	Male	Female	Total
	(n = 378)	(n = 461)	N = 839
	Mean (SD)		
Age (years)	18.81 (0.58)	18.76 (0.62)	18.78 (0.60)
Weight (kg)**	71.02 (14.46)	62.59 (15.59)	66.4 (15.65)
Height (cm)**	176.65 (6.57)	163.61 (6.13)	169.48 (9.06)
Body Mass Index	22.73 (4.37)	23.35 (5.60)	23.08 (5.09)
Waist Circumference (cm)	75.17 (11.11)	73.99 (12.10)	74.52 (11.67)
Hip Circumference (cm)*	94.36 (9.02)	96.54 (11.13)	95.56 (10.29)
Systolic BP (mmHg)**	113.93 (10.53)	107.43 (8.92)	110.36 (10.20)
Diastolic BP (mmHg)**	69.40 (10.38)	66.90 (9.26)	68.03 (9.85)
Fasting Glucose (mmol/l)**	4.72 (0.56)	4.44 (0.37)	4.57 (0.49)
HDL (mmol/l)**	1.14 (0.24)	1.25 (0.30)	1.20 (0.28)
Triglycerides (mmol/l)*	0.61 (0.27)	0.56 (0.26)	0.58 (0.26)

	Proportion % (SE)		

Physical Activity Level			

Low (No activity)**	18.0 (2.0)	47.1 (2.3)	34.0 (1.6)

*Moderate (≤ 3.5 hours)*	45.2 (2.6)	39.7 (2.3)	42.3 (1.7)

High (>3.5 hours)**	36.8 (2.5)	13.2 (1.6)	23.8 (1.5)

Hypertension	2.1 (0.7)	0.9 (0.4)	1.4 (0.4)

SD = Standard deviation; BP = Blood Pressure; HDL = High density lipoprotein cholesterol

*p < 0.05, **p < 0.001

Only one participant had diabetes mellitus and was on medication for diabetes. None of the participants were currently on medication for hypertension

We also compared available information for the 732 participants who were in the targeted sample but were not enrolled to the 902 who were enrolled. Of the 732 persons not enrolled 68 refused to participate, 143 had migrated out of Jamaica, 7 had died, 2 were incarcerated and 512 who failed to keep their appointment, did not commit to an appointment or could not be contacted. A higher proportion of targeted males were not enrolled (48% male vs. 42% female, p = 0.012). There were no differences in the parish of residence or rural vs. urban addresses for those enrolled compared to those not enrolled.

### Prevalence of the metabolic syndrome and its components

The prevalence of the full metabolic syndrome, two or more components, one or more components and the individual metabolic syndrome components are shown in Table 2. The overall prevalence of the metabolic syndrome was 1.2% (95% confidence interval [95%CI] 0.5%-1.9%). This prevalence was the same when the 2005 IDF criteria were used, while the prevalence using the 2005 revised ATP-III criteria was 1.0% (95%CI 0.3%-1.6%). A little over half (54%) of participants had at least one component and 15.5% had two or more components. There were no statistically significant sex differences in the prevalence of the metabolic syndrome; however females were more likely than males to have at least one or two criteria. Of the individual metabolic syndrome components, low HDL had by far the highest prevalence, 46.8%. Central obesity had the second highest prevalence 16.0%. Almost 7% of participants had elevated blood pressure. High triglycerides and impaired fasting glucose had very low prevalence, 0.6% and 1.2%, respectively. Impaired fasting glucose and elevated BP were more common in males while females had higher prevalence of central obesity and low HDL.

**Table 2:** Prevalence of the metabolic syndrome and components among all study participants and by sex

Syndrome & components	Male (n = 378)		Female (n = 461)		Total (N = 839)	
	% (95%CI)		% (95%CI)		% (95%CI)	
**Occurrence of the Metabolic Syndrome and combinations of its components**						

**Metabolic Syndrome (≥ 3 components)**	**0.5**	**(0.2-1.3)**	**1.7**	**(0.5-2.9)**	**1.2**	**(0.5-1.9)**
≥ 2 Metabolic Syndrome Components***	8.5	(5.7-11.3)	21.3	(17.5-25.0)	15.5	(13.0-17.9)
≥ 1 Metabolic Syndrome Components***	38.6	(33.7-43.5)	67.5	(63.2-71.7)	54.4	(51.1-57.8)

**Individual Components**						

WC***	5.3	(3.0-7.5)	24.7	(20.8-28.6)	16.0	(13.5-18.5)
High BP***	10.8	(7.7-14.0)	3.3	(1.6-4.9)	6.7	(5.0-8.4)
IFG***	2.1	(0.6-3.6)	0.4	(0-1.0)	1.2	(0.4-1.9)
Low HDL***	28.8	(24.3-33.4	61.6	(57.2-66.0)	46.8	(43.5-50.2)
High Triglycerides	0.5	(0-1.2)	0.6	(0-1.3)	0.6	(0.1-1.1)

WC = elevated waist circumference/central obesity; BP = Blood Pressure; IFG = Impaired Fasting Glucose; HDL = High density lipoprotein cholesterol

* p < 0.05 ** p < 0.01 ***p < 0.001 for male: female difference in proportion

### Association between metabolic syndrome components and socioeconomic status

Table 3 shows the prevalence of the metabolic syndrome components by levels of the socioeconomic status markers. There was a significant association between central obesity and household head's occupation and parental education. Central obesity had a higher prevalence among participants who lived in households headed by semi-skilled/unskilled persons (21.8%) compared with those headed by highly skilled persons (12.4%). There was a significant trend for higher prevalence of central obesity among those in the lower occupation groups (p = 0.009). Central obesity also had a higher prevalence among participants whose parents/guardian had primary/all-age education (28.4%) compared to those with tertiary education (12.5%). There was a significant trend (p = 0.002) for higher prevalence of central obesity among the lower education groups. Elevated glucose was associated with household tenure (p = 0.017), but there was no trend. None of the other metabolic syndrome components showed any significant associations with household tenure.

**Table 3:** Prevalence of the metabolic syndrome components by levels of socioeconomic status markers

Occupation of Household Head						
	Highly Skilled	Skilled	Semi/Unskilled	Other	P value (association)	P value* (trend)
**WC**	**12.43**	**13.55**	**21.84**	**22.81**	**0.013**	**0.009**
High BP	7.03	6.39	7.28	5.26	0.942	0.908
IFG	1.08	1.28	0.49	3.51	0.319	0.541
Low HDL	43.78	45.27	53.40	43.86	0.184	0.052
High TG	1.62	0.51	0.00	0.00	0.179	0.047

**Parental Education**						
	Tertiary	Secondary	Primary/All Age	Don't Know	P value (association)	P value (trend)*

**WC**	**12.50**	**14.08**	**28.43**	**18.18**	**0.002**	**0.002**
High BP	6.25	6.68	6.86	7.27	0.988	0.818
IFG	0.96	1.19	0.98	1.82	0.919	0.927
Low HDL	48.56	47.26	48.04	40.91	0.596	0.870
High TG	1.44	0.24	0.98	0.00	0.228	0.369

**Housing Tenure**						
	Owned by Parent	Family Owned	Rented	Other	P value (association)	P value (trend)*

WC	15.78	14.79	15.92	21.05	0.733	0.992
High BP	6.31	8.28	6.97	3.51	0.629	0.667
**IFG**	**0.73**	**2.96**	**0.00**	**3.51**	**0.017**	**0.723**
Low HDL	45.87	45.56	50.25	45.61	0.744	0.349
High TG	0.49	1.18	0.50	0.00	0.695	0.852

*Test for Trend excludes other/don't know categories; WC = elevated waist circumference; BP = Blood pressure; IFG = Impaired Fasting Glucose; HDL = High density lipoprotein cholesterol; TG = triglycerides

### Multivariate analyses

Logistic regression models were used to assess the relationship between the prevalence of the metabolic syndrome and its individual components and measures of socioeconomic status. There were no significant associations with the overall prevalence of the metabolic syndrome and the socioeconomic status measures used. Of the individual components central obesity was associated with occupation of the household head and parental education, while low HDL was associated with occupation of the household head only. There were no significant associations in logistic regression models for elevated blood pressure, elevated glucose or triglycerides using the metabolic syndrome criteria.

After adjusting for age, sex and BMI (overweight vs. normal) the odds ratio for low HDL comparing semi-skilled/unskilled to the highly skilled group was 1.55 (95%CI 1.01-2.38, p = 0.046). Associations comparing the skilled and 'other' occupational categories to the highly skilled category were not significant (data not shown).

Table 4 shows the odds ratios, derived from logistic regression models, for the association between central obesity and levels of education and occupation among male and female study participants. The final model used sex specific estimates because sex was a statistically significant effect modifier of the association between central obesity and occupational or educational status. Adjustments were made for hip circumference but age was excluded from the final models as age did not improve the model based on the likelihood ratio tests. Increased odds of central obesity were found in participants with lower levels of occupation and education among women but not among men. In contrast, lower education and occupation were associated with reduced odds for central obesity among men, but was not statistically significant.

**Table 4:** Odds ratios for association between central obesity among male and female participants by level of parental occupation and education

SES Category	Odds Ratio	95%CI	P Value
**Occupation**			

**Male**			
Highly Skilled	Reference group		
Skilled	0.44	0.08-2.28	0.328
Semi/Unskilled	0.64	0.10-4.00	0.630
Other	3.39	0.35-32.71	0.290
**Female**			
Highly Skilled	Reference group		
Skilled	2.55	0.99-6.57	0.054
Semi/Unskilled	3.37	1.22-9.29	**0.019**
Other	4.67	1.17-18.55	**0.029**

**Education**			

**Male**			
Tertiary	Reference group		
Secondary	0.50	0.11-2.23	0.363
Primary/All Age	0.01	0-7.32	0.157
Don't Know	1.44	0.21-9.82	0.704
**Female**			
Tertiary	Reference group		
Secondary	1.72	0.74-4.01	0.205
Primary/All Age	6.14	2.05-18.40	**0.001**
Don't Know	4.61	1.47-14.39	**0.009**

Estimates derived from sex-specific logistic regression models as there was evidence of interaction by sex in the relationship between central obesity with occupation and education.

Adjustment was made for hip circumference.

Age was excluded from the model after model assessment (likelihood ratio test) showed that it did not improve model.

## Discussion

The prevalence of metabolic syndrome among Jamaican youth was estimated at only 1.2% using the 2009 consensus criteria; however a large proportion of participants (54%) exhibited at least one metabolic syndrome component. Of the individual components, low high density lipoprotein cholesterol (HDL) was the most common (46.8%) while impaired fasting glucose and elevated triglycerides were rare (1.2% and 0.6%, respectively). There were significant sex differences in the prevalence of all the metabolic syndrome components except elevated triglycerides, but there was no sex difference in the overall prevalence of the metabolic syndrome, probably related to the small number of cases in the sample.

Socioeconomic status was not associated with the overall prevalence of the metabolic syndrome but central obesity was more common among persons in the lower categories of occupation and education among women. Participants in the semi-skilled/unskilled occupation group were more likely to have low HDL.

### Comparison of findings with the current literature

The prevalence of the metabolic syndrome found in this study is lower than that reported in most studies in the published literature. Among adolescents aged 12-19 years in the United States, Duncan and colleagues reported a prevalence of 4.2% using NHANES III (1988-1992) data and 6.4% using NHANES 1999-2000 data [14]. Other reports of population based metabolic syndrome prevalence estimates among adolescents or young adults have ranged from 1.4% in Japanese young men (mean age 15 years) [17] to 10.1% among urban adolescents (10-19 years old) in Iran [15]. Of note however, the prevalence of the metabolic syndrome among non-Hispanic blacks in Duncan's study was estimated at 2.0% using NHANES III (1988-1992) data and 5.1% using NHANES 1999-2000 data. This suggests that the prevalence of the metabolic syndrome may be lower in youth of African descent compared to Caucasians or Hispanics. This was further supported by Johnson et al who reported a prevalence of 4.0% among US black youth 12-19 years old observed between 2000 and 2006, compared to 8.9% among whites and 11.2% among Hispanics [18]. The lower prevalence in Caribbean black youth compared to US black youth may reflect the difference in stage of development via differences in diet, exercise and other lifestyle factors. No study reporting on the prevalence of the metabolic syndrome among adolescents or young adults in Africa was found in our literature search, however one study [40] reporting on the prevalence of the metabolic syndrome among adults in Cameroon reported very low prevalence of the metabolic syndrome in both rural and urban populations. Using the IDF criteria prevalence of the metabolic syndrome was 1.5% and 1.2% among urban women and men respectively; while among the rural population prevalence among women was 0.3% and it was absent in men.

With regards to the prevalence of the individual components of the metabolic syndrome the high prevalence of low HDL and central obesity is consistent with findings from another study among Jamaican adults, which reported prevalence of 45.5% for low HDL and 45.8% for central obesity, respectively [41]. Similarly, hypertriglyceridemia had the lowest prevalence in this study. Among black adolescents in the USA low HDL and central obesity were also the most common metabolic syndrome abnormality while impaired fasting glucose was the least common [14].

In this report there were no significant associations between the measures of socioeconomic status studied and the prevalence of the metabolic syndrome. This may have been due to the very small number of participants with the metabolic syndrome resulting in the study being under powered to detect differences. Only a few studies looking at the prevalence of metabolic syndrome in youth evaluated the impact of socioeconomic status, and findings so far are inconsistent [29,42-44]. For example, Loucks and colleagues [29] found that while the metabolic syndrome was associated with socioeconomic position in females aged 25-65 years, the associations were weak among adolescents, males, and older participants. Ozaki and colleagues [44] however found that although there was no association between father's occupation and metabolic syndrome prevalence, participants who attended lower grade schools were significantly more likely to meet the metabolic syndrome criteria.

Despite the lack of association between socioeconomic status and metabolic syndrome overall, associations were found with the prevalence of central obesity and low HDL. Of note, there was significant interaction with sex in the association between central obesity and socioeconomic status, where the statistically significant inverse relationship seen in females only with a non-significant positive relationship among males. Associations between central obesity and markers of socioeconomic status have also been reported in other studies. Goodman and colleagues reported that low parent education was associated with higher waist circumference among adolescents from the mid-western United States [45]. Among Cameroon women however, no significant association was found with occupation level, while men had higher prevalence of abdominal obesity with higher occupation [46]. Data from Korea also show significant sex differences in the relationship between abdominal obesity and socioeconomic status [47]; women showed significantly decreased odds for abdominal obesity with higher education while men showed a non-significant increased odds for abdominal obesity with higher education, a pattern similar to our study. These studies highlight the different patterns in the association between markers of socioeconomic status and abdominal obesity and underscore the need for further research in this area. The findings in this study may also reflect the contrasting associations between measures of SES and CVD risk factors in developed and lesser developed countries. In the more developed societies CVD risk factor burden is inversely related to SES while a positive association may be seen in less developed countries [22,23]. The direction of the association appears to be related to stage of the epidemiological transition being experienced in the specific countries [23].

### Strengths and limitations of study

This study was done using a population based sample of youth from urban Jamaica and should therefore be representative of the urban population of similarly aged Jamaicans. The response rate however was less than desired as only 75% of contacted participants and 55% of targeted participants were enrolled into the study. This introduces the possibility of selection bias and therefore limits the generalizabilty of the findings. This study however represents the best estimates of prevalence of metabolic syndrome among youth from the region and among blacks in the developing world as there are no other published studies on the subject.

Measurements made during the study followed standardized procedures thus limiting the likelihood of measurement error and misclassification. However since determination of socioeconomic status was based on questionnaire data enquiring about parental socioeconomic status from youth without validation by parents, some participants may have had inaccurate information on these questions, and thus could result in misclassification. In addition, occupational categories required coding of the stated occupation into categories after data collection which could also introduce some misclassification. We have no reason to believe that any misclassification would introduce bias (differential misclassification) and our estimates of differences are likely to be more conservative than extreme and are likely to be valid.

Participants with missing values were excluded from the analysis to ensure that all analyses were done on the same data. The proportion with missing values were relatively small (7%) and there were no differences in baseline characteristics for those excluded compared to those included in the analysis. It is therefore unlikely that any bias resulted from the exclusion of persons with missing data.

### Implications

This study is the first to report on the prevalence of the metabolic syndrome among youth in the Caribbean and reports a relatively low prevalence of metabolic syndrome among predominantly black Jamaican youth. It raises the issue of both racial/ethnic differences and stage of economic development (westernization) in metabolic syndrome prevalence when estimates are compared to reports from the United States (whites and blacks) and Cameroon.

## Conclusions

The metabolic syndrome is relatively uncommon among Jamaican youth when compared to prevalence reported from other western populations and in view of other reports suggest differences related to both westernization as well as race/ethnicity in the prevalence of the syndrome. Prevalence of central obesity and low HDL were relatively high. Central obesity was associated with socioeconomic status in females only. There were no associations between the overall prevalence of the metabolic syndrome and socioeconomic status in this study.

## Competing interests

The authors declare that they have no competing interests

## Authors' contributions

**TSF **- supervised field activities for data collection, designed the data-analysis strategies, lead the data analysis, drafted and critically reviewed the manuscript. **MKTR **- contributed to data collection, data analysis and critical review of the manuscript. **NOMY **- contributed to data collection, data analysis and critical review of the manuscript. **JMKM **- contributed to data collection, data interpretation and critical review of the manuscript. **MSV **- contributed to the design of the study and critical review of the manuscript. **DA **- contributed to the design of the study and critical review of the manuscript. **JVDB **- contributed to data collection and quality assurance and control, and critical review of the manuscript. **RJW **- conceived and designed the study and directed its implementation including quality assurance and control, contributed to the data analysis strategies and data interpretation, critically reviewed drafts of the manuscripts and is its guarantor. All authors have read and approved the final manuscript.

## Pre-publication history

The pre-publication history for this paper can be accessed here:

http://www.biomedcentral.com/1471-2458/10/307/prepub
